# Synergistic effects of transcutaneous vagus nerve stimulation and inhibitory control training on electrophysiological performance in healthy adults

**DOI:** 10.3389/fnins.2023.1123860

**Published:** 2023-03-09

**Authors:** Chunchen Wang, Lingwei Zeng, Xinsheng Cao, Jing Dai, Yang Liu, Zhijun Gao, Yilong Qin, Lin Yang, Hang Wang, Zhihong Wen

**Affiliations:** ^1^Department of Aerospace Medicine, Fourth Military Medical University, Xi’an, China; ^2^Department of Medical Psychology, Air Force Medical University, Xi’an, China

**Keywords:** transcutaneous vagus nerve stimulation (tVNS), inhibitory control training, EEG, N2, alpha oscillation, inhibitory control enhancement

## Abstract

Transcutaneous vagal nerve stimulation (tVNS) is a non-invasive nerve stimulation technique that exerts a positive “exogenous” online neuromodulatory effect on inhibitory control (IC). Additionally, IC training (ICT) is an effective approach for enhancing IC *via* the “endogenous” activation of brain regions implicated in this process. The aim of the present study was to examine the synergistic effects of tVNS and ICT on IC enhancement. For this, we measured the changes in neural activity in frontal, fronto-central, and central regions in the time domain of the N2 component and the frequency domain of alpha power during the stop signal task. A total of 58 participants were randomly divided into four groups that received five sessions of either ICT or sham ICT with either online tVNS or sham tVNS. No differences in N2 amplitude were detected after any of the interventions. However, N2 latency shortened after tVNS + ICT in frontal, fronto-central, and central regions. N2 latency shortened after the intervention of sham tVNS + ICT in frontal region. Moreover, alpha power after tVNS + ICT intervention was larger than those of the other interventions in frontal, fronto-central, and central regions. The obtained electrophysiological data suggested that combining tVNS with ICT has synergistic ameliorative effects on IC, and provide evidence supporting the IC-enhancing potential of tVNS combined with ICT.

## 1. Introduction

Transcutaneous vagus nerve stimulation (tVNS) is a non-invasive brain stimulation technique (Ridgewell et al., 2021) that has been suggested as a potential method by which to modulate inhibitory control (IC) *via* the activation of the locus coeruleus-noradrenergic (LC-NE) pathway (Warren et al., 2019; Rodenkirch et al., 2022). Studies on tVNS have demonstrated widespread activity in expected vagal projection areas (e.g., locus coeruleus, prefrontal cortex) (Rings et al., 2021). Additionally, tVNS was reported to produce a significantly greater BOLD signal in the frontal cortex based on a concurrent tVNS/fMRI analysis (Badran et al., 2018). tVNS can also modulate specific markers of cortical excitability in participants undergoing transcranial magnetic stimulation combined with electroencephalography (TMS-EEG) (Capone et al., 2015; Mertens et al., 2022) and has been associated with increased activity in the frontal lobe, likely as a consequence of vagal afferents transducing signals to higher-order brain centers (Rajiah et al., 2022). Peripheral autonomic nervous system activity and that relative changes in PFC oxygenation contribute to these effects as quantified using functional near-infrared spectroscopy (fNIRS) by the research about tVNS on cognitive function (Hoper et al., 2022). The NE system has been reported to play a varied and complex role in executive function (Usher et al., 1999; Slater et al., 2022; Tomassini et al., 2022). Moreover, recent studies have reported that tVNS can induce remarkable changes in IC, as revealed by electrophysiological data, such as event-related potentials (ERP). For example, tVNS has been found to result in a decrease in the amplitude of N2 (Pihlaja et al., 2020) and an increase in that of P3 (Ventura-Bort et al., 2018), which are markers of cognitive control. tVNS was also reported to induce an increase in the metrics of electroencephalogram (EEG) microstate *A* mean duration (Ricci et al., 2020) and enhance the frontal midline theta power spectrum in a go/no-go task (Keute et al., 2019). These findings demonstrate the potential of tVNS to modulate IC. However, the LC-NE activation-associated increases in brain norepinephrine levels induced by tVNS are transient and return to baseline levels when tVNS is stopped (Van Leusden et al., 2015). These findings may explain why resting beta and gamma oscillations, measured using magnetoencephalography, are not affected by tVNS (Keute et al., 2021). This indicates that tVNS, as “exogenous” online neuromodulation, might facilitate the activation of neural networks that are associated with IC.

Based on the theory of neuroplasticity, cognitive training has been proposed as an effective approach for enhancing IC (IC training; ICT). ICT has been defined as a repeated practice that adapts one or multiple standardized paradigms to specifically target IC in cognitive functioning (Elmasry et al., 2015). Previous research showed that ICT can enhance IC in healthy individuals, which is attributable to the “endogenous” activation of specific brain regions that are closely associated with IC during practice periods (Millner et al., 2012). However, the limitations of ICT include poor transfer to non-trained tasks, a lack of continuous practice motivation, and increased mental workload due to long-term repeated practice.

Given the different neurological mechanisms underlying the effects of tVNS and ICT on IC enhancement, our group developed a novel intervention approach involving tVNS combined with ICT. In a recent behavioral study, we revealed that this approach can cause a training effect and transfer effect and achieve IC enhancement in healthy individuals (Wang et al., 2022). However, the behavioral indicator of IC was not sufficient to evaluate the efficacy of the combined tVNS + ICT intervention, as participants were instructed not to produce a behavioral response to some types of stimuli. ERP research has provided another reliable electrophysiological measure of neural activity associated with IC enhancement.

The amplitude and latency of N2, an early ERP component, have been demonstrated to be associated with IC. Electrophysiological studies using the stop signal task (SST) or the go/no-go task have found a significant fronto-central N2 component in signal or no-go trials compared to go trials (Groom and Cragg, 2015; Dippel et al., 2017). Moreover, the signal- or no-go-N2 component has been reported to reflect an individual’s detection (Chmielewski and Beste, 2017) or monitoring of conflict (Raud et al., 2020) and initiated response, which may reflect inhibition processes (Ghin et al., 2022). Anterior fronto-central N2 latency has been reported to be longer in adults with attention deficit hyperactivity disorder (ADHD) (Fisher et al., 2011) and those with sleep deprivation (Kusztor et al., 2019). Additionally, older people with mild cognitive impairment have been reported to have a longer N2 latency than healthy younger people (Chiang et al., 2018; Cid-Fernandez et al., 2019). These findings indicate that N2 latency may serve as a neural marker of IC and that anterior cortex-evoked N2 amplitude is strongly and positively correlated with response inhibition.

In addition to ERP, event-related oscillations (EROs) are a direct measure of neural activity that is time-locked to IC. EROs are typically analyzed by decomposing the event-related EEG signal into phase and magnitude information over a range of frequencies (Pandey et al., 2016). The high spatial and temporal resolution of EROs enables time-frequency analysis of regional brain activity to investigate neural dynamics in cognitive processes (e.g., IC). Cortical neural oscillations in the EEG alpha band (8–13 Hz) have been reported to reflect the most basic cognitive processes (Klimesch, 2012; Foster and Awh, 2019), as well as be linked with the suppression or inhibition of task-irrelevant information (Jensen and Mazaheri, 2010; Wiesman et al., 2019; van Zoest et al., 2021) and increased signal-to-noise ratio of activity within the cerebral cortex (Vaden et al., 2012; Hwang et al., 2016). Several studies have proposed that there exists an alpha-associated inhibitory gating process for interfering information in the frontal cortices. An increase in alpha activity may reflect inhibitory gating processes, whereby increased alpha power-blocking oscillation activity could block interfering or irrelevant signals to improve information processing efficiency and strengthen the dynamic functional responsiveness of brain networks (Konjusha et al., 2022). In cognitive control paradigms such as the SST or the go/no-go task, response inhibition after a stop signal or no-go stimulus has been associated with increased alpha power (Schmiedt-Fehr et al., 2016). However, people with alcohol addiction who have weak IC display significantly lower evoked alpha power compared with healthy adults for the no-go stimulus (Händel et al., 2011; Pandey et al., 2016).

Therefore, the aim of the present study was to investigate changes in neural activity in distinct brain regions following tVNS combined with ICT, including the time domain of ERP (signal-N2) and the frequency domain of EROs (alpha power), which reflect IC performance. We have previously demonstrated that this novel approach of tVNS combined with ICT can improve IC using behavioral analysis (Wang et al., 2022). Here, to further evaluate the synergistic effect of tVNS combined with ICT, we extend this investigation to neurophysiological performance. We hypothesized that IC performance, reflected by frontal and central neural activity, would be enhanced using tVNS combined with ICT.

## 2. Materials and methods

### 2.1. Participants

A total of 58 male undergraduate students (M_age_ = 19.5 years, SD = 0.7 years) were recruited *via* leaflets. According to the actual sample size, the *post-hoc* power was calculated as 0.88 using G*Power software (ver. 3.1.9.7.2; Heinrich-Heine-Universität Düsseldorf, Düsseldorf, Germany) (Kang, 2021). All participants were right-handed and had not previously participated in similar cognitive intervention research. Participants underwent a Web-based screening questionnaire before the experiment to ensure that they met the inclusion/exclusion criteria. The exclusion criteria included color blindness, a history of any psychological or neurological disorder, brain trauma or surgery, heart-related diseases, and adult ADHD assessed using the adult ADHD self-reporting scale (Kessler et al., 2005). A total of 60 participants passed the initial screening and were enrolled in the experiment. Two withdrew before completing the experiment due to personal schedule conflicts. The remaining 58 participants were allocated to one of the four following groups using a randomized single-blinded method: A tVNS + ICT group (*n* = 14), a sham tVNS + ICT group (*n* = 15), a tVNS + sham ICT group (*n* = 15), and a sham tVNS + sham ICT group (*n* = 14). All the participants provided written informed consent before participation and received 100 RMB/h as compensation for completing the experimental tasks efficiently. All procedures in the study were carried out in accordance with the Declaration of Helsinki and were approved by the Medical Ethics Committee of the Air Force Medical University (NO.KY20213079-1).

### 2.2. Apparatus and procedure

**FIGURE 1:**
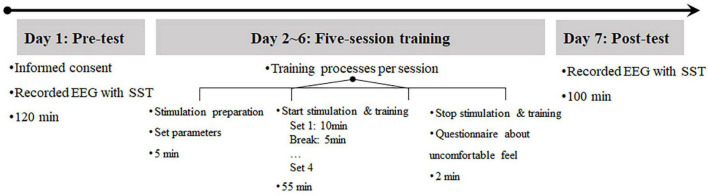
Overview of the experimental procedure and timeline in the study.

Each participant was required to complete three sequential experimental phases, including a pre-test, a five-session training, and a post-test. The experimental schedule of each participant (Figure 1) lasted for approximately two weeks. The pre-test was completed 1–2 days before the experiment began. The post-test was completed 1–2 days after the training phase. The five-session training phase consisted of five sessions of combined simultaneous tVNS (or sham tVNS) and ICT (or sham ICT). The training frequency was once a day, and each training session lasted approximately 60 min, during which each participant was required to complete four sets of combined simultaneous tVNS (or sham tVNS) and ICT (or sham ICT). Each ICT (or sham ICT) set comprised 240 trials, with a 5-min rest period between training sets. tVNS applied during ICT also included five sessions. The frequency of tVNS was the same as that for ICT, and the duration of each set of tVNS was equal to the duration of each set of ICT, which was approximately 60 min.

### 2.3. Intervention protocol

During the tVNS, two electrodes were placed at the cymba concha of the left ear. Based on previous tVNS studies, the parameters of the stimulation device (tVNS501, Rishena, Changzhou, China) were set to continuously deliver electrical stimulations with a pulse width of 200–300 μs at 25 Hz and a biphasic pulse interval of 30 s ON and 30 s OFF. The stimulus intensity of the tVNS varied between individuals and was set to the average level, which was defined by the level above the detection threshold but below the pain perception threshold (Ellrich, 2011; Pihlaja et al., 2020). All participants reported a strong “tingling” sensation to the stimulation condition but no uncomfortable feelings. For the sham tVNS, the electrodes were placed on the left earlobe. Except for the different electrode positions, all parameters were identical between the tVNS and sham tVNS groups.

Each set of the SST was adapted to the ICT paradigm and included 180 go trials and 60 stop trials. The time of stop-signal delay (SSD) in the SST was adjusted according to the performance of the participants on the stop trials; the initial SSD was 250 ms, and after reacting correctly to a stop trial, the SSD increased by 50 ms, whereas after reacting incorrectly to a stop trial, the SSD decreased by 50 ms. The SSD ranged from 0 to 750 ms. Performance on the SST was assessed using the stop-signal reaction time, which was calculated as the average difference between the reaction time (RT) of go trials and the SSD. A lower stop-signal reaction time reflected stronger response inhibition. The exclusion criteria in the SST analysis included a RT of less than 150 ms or more than 1000 ms on go trials or stop-failure trials, and a mean RT of stop-failure trials longer than the mean RT of go trials and *p*_(*respond|signal*)_ lower than 0.25 or higher than 0.75 (Congdon et al., 2012; Verbruggen et al., 2019). All participants were required to complete 5 sessions of ICT. For the sham ICT paradigm, the simple reaction task of each set comprised 240 trials, and trials of the “training session of sham ICT” with an accuracy level below 90% were excluded. Other parameters of the simple reaction task were the same as those of the SST.

### 2.4. Electrophysiological recording and data analysis

During the SST at both the pre- and post-test phases, continuous EEG data were recorded using a 32-channel Grael EEG amplifier (Compumedics Germany GmbH, Germany) that was configured according to the international 10–20 placement system with the midline reference located at the vertex between Cz and CPz, and the grounding electrode located near the area of Fz. The electrooculogram was recorded *via* two bipolar electrodes placed at the lateral canthus of each eye (horizontal electrooculogram) and above and below the left eye (vertical electrooculogram). The EEG data were sampled at 1024 Hz using Curry 8.05 Recorder software (Compumedics Neuroscan, Germany), and the impedance of each electrode was kept below 20 kilohms.

The recorded EEG data were preprocessed offline using the EEGLAB toolbox (v. 14.1.1) in MATLAB R2020b (The MathWorks, Inc., MA, USA). Preprocessing included removing non-brain and invalid EEG channels from subsequent analysis, re-referencing to the average of left and right mastoids, band-pass filtering (1–30 Hz), down-sampling to 128 Hz, and removing eye blinks, saccades, muscle artifacts, swallowing, or other noise artifacts using independent component analysis-based correction. Epochs of 1000 ms were extracted from -200 to 800 ms relative to stimulus onset for each trial, followed by baseline correction to a pre-stimulus interval of 200 ms, and epochs exceeding the voltage threshold of ±80 μV in amplitude at any channel were excluded from subsequent analysis.

In the present study, frontal (F11, F7, F3, FZ, F4, F8, F12), fronto-central (FC3, FCZ, FC4), and central (C3, CZ, C4) regions were the regions of interest (ROIs). The peak amplitudes and latencies of the N2 based on stop signal trials of the SST (signal-N2) were defined as the peak negative signal. The corresponding peak latency in the time windows from 100 to 300 ms after stimulus onset, and the power spectral density (PSD) of the alpha band with time-locking events (stop signal trials of the SST) were calculated using fast Fourier transform in the 800-ms time window after stimulus onset in the ROIs (frontal, fronto-central, and central regions).

### 2.5. Statistical analysis

The ERP parameters (N2 amplitude and latency) and EROs parameter (PSD of the alpha power) were analyzed separately using repeated measures analysis of variance (rmANOVA), executed in SPSS 25 (IBM Inc., New York, NY, USA) with 2 phases (pre- and post-test), 4 groups (tVNS *vs*. sham tVNS, ICT vs. sham ICT) in each ROI (frontal, fronto-central, and central regions). The main effects of phase and group and the interaction effects were analyzed. To further explore the within-group (pre- and post-test) and between-group differences at each phase, simple effects analysis with *post-hoc* Bonferroni correction was performed. Additionally, between-group differences in baseline variables, such as age and years of education, were assessed using one-way ANOVA.

Significance was set at a value of α of 0.05. The effect size was estimated using partial eta-squared (η_*p*_^2^). Data were analyzed using SPSS software (version 25, IBM Inc., New York, NY, USA). Descriptive statistics are reported as means ± standard deviation.

## 3. Results

### 3.1. Baseline parameters

Descriptive statistics, including the means and standard deviations, are reported in Table 1. There were no significant between-group differences in age [*F*_(3,54)_ = 0.44, *p* = 0.73] or years of education [*F*_(3,54)_ = 0.11, *p* = 0.95] (Table 1). ERP and EROs data in each ROI are presented in Table 1 and the results are shown in Table 2. For the frontal region, the results revealed a significant main effect of phase on signal-N2 latency and amplitude as well as alpha power. There was no significant main effect of group and no phase × group interaction effect on signal-N2 latency and amplitude. However, there was a significant main effect of group and a significant phase × group interaction effect on alpha power. For the fronto-central region, the results revealed a marginally significant main effect of phase on signal-N2 latency and alpha power, a marginally significant main effect of group on alpha power, and a significant phase × group interaction effect on signal-N2 latency and alpha power. For the central region, there was a significant phase × group interaction effect on alpha power.

**TABLE 1:** Mean (standard deviation) of baseline variables including age and years of education, N2 component, and alpha power in ROIs during pre- and post-test.

Group	Age (years)	Edu (years)	Region	Phase	N2-Lat (ms)	N2-Amp (μ V)	PSD-alpha (db)
tVNS + ICT (*n* = 14)	19.50 (0.65)	13.60 (0.27)	*F*	*Pre*-	235.89(39.59)	−3.67(3.78)	17.23(0.80)
				*Post*-	215.00(25.45)**	−3.89(1.93)	18.62(0.88)**
			*FC*	*Pre*-	217.26(43.52)	−5.13(7.32)	19.18(1.38)
				*Post*-	189.36(18.11)**	−5.19(3.37)	20.36(0.97)**
			*C*	*Pre*-	217.45(35.05)	−4.49(7.09)	18.70(1.61)
				*Post*-	199.78(24.20)*	−5.78(3.35)	19.88(1.17)**
sham tVNS + ICT (*n* = 15)	19.53 (0.83)	13.62 (0.30)	*F*	*Pre*-	232.51(28.89)	−3.17(3.08)	17.51(1.41)
				*Post*-	217.34(27.13)*	−4.03(2.68)	17.11(1.38)^+^
			*FC*	*Pre*-	206.60(33.02)	−3.62(4.43)	19.09(1.34)
				*Post*-	204.86(21.67)	−4.99(3.20)	18.71(1.26)
			*C*	*Pre*-	209.20(26.37)	−3.12(4.66)	18.69(1.28)
				*Post*-	206.25(19.96)	−4.93(3.34)	18.47(1.29)
tVNS + sham ICT (*n* = 15)	19.33 (0.72)	13.54 (0.51)	*F*	*Pre*-	229.09(29.41)	−3.35(3.07)	16.86(1.42)
				*Post*-	227.98(23.16)	−5.27(2.84)*	16.20(0.80)**
			*FC*	*Pre*-	201.74(35.76)	−4.49(5.67)	18.96(1.56)
				*Post*-	195.31(18.93)	−6.25(5.47)	18.22(0.82)**
			*C*	*Pre*-	213.72(39.59)	−4.06(5.93)	18.73(1.70)
				*Post*-	198.26(18.59)^+^	−5.05(5.94)	17.72(0.81)**
Sham tVNS + sham ICT (*n* = 15)	19.64 (0.75)	13.57 (0.53)	*F*	*Pre*-	233.58(19.21)	−5.03(3.52)	16.82(1.24)
				*Post*-	229.03(26.37)	−5.45(3.21)	17.57(1.21)**
			*FC*	*Pre*-	202.57(26.46)	−8.42(5.01)	18.73(1.33)
				*Post*-	206.47(29.70)	−8.18(4.02)	19.61(1.25)**
			*C*	*Pre*-	202.94(26.15)	−8.16(4.74)	18.26(1.19)
				*Post*-	207.03(26.08)	−7.57(3.93)	18.78(1.35)*

Edu, years of education; N2-Lat, N2 latency; N2-Amp, N2 amplitude; PSD-alpha, PSD of alpha power; F, frontal region; FC, fronto-central region; C, central region. **p < 0.01, *p < 0.05, ^+^*p* < 0.08.

**TABLE 2:** Summary of rmANOVA results for signal-N2 latency and amplitude, alpha power in ROIs.

Effect	N2-Lat			N2-Amp			PSD-alpha		
	**F**	**FC**	**C**	**F**	**FC**	**C**	**F**	**FC**	**C**
	***F*(η*_*p*_^2^*)**	***F*(η*_*p*_^2^*)**	***F*(η*_*p*_^2^*)**	***F*(η*_*p*_^2^*)**	***F*(η*_*p*_^2^*)**	***F*(η*_*p*_^2^*)**	***F*(η*_*p*_^2^*)**	***F*(η*_*p*_^2^*)**	***F*(η*_*p*_^2^*)**
phase	7.98** (0.13)	3.95^+^ (0.07)	3.62 (0.06)	4.15* (0.07)	1.25 (0.02)	1.99 (0.04)	6.05* (0.10)	3.49^+^ (0.06)	0.92 (0.02)
group	0.22 (0.01)	0.23 (0.01)	0.07 (<0.01)	1.14 (0.06)	2.34 (0.12)	2.10 (0.11)	3.97* (0.18)	2.65^+^ (0.13)	1.95 (0.13)
phase × group	1.54 (0.08)	2.87* (0.14)	1.49 (0.08)	0.83 (0.04)	0.54 (0.03)	0.68 (0.04)	18.83** (0.51)	14.17** (0.44)	15.74** (0.47)

N2-Lat, N2 latency; N2-Amp, N2 amplitude; PSD-alpha, PSD of alpha power; F, frontal region; FC, fronto-central region; C, central region. **p < 0.01, *p < 0.05, ^+^*p* < 0.08.

### 3.2. N2 component

**FIGURE 2:**
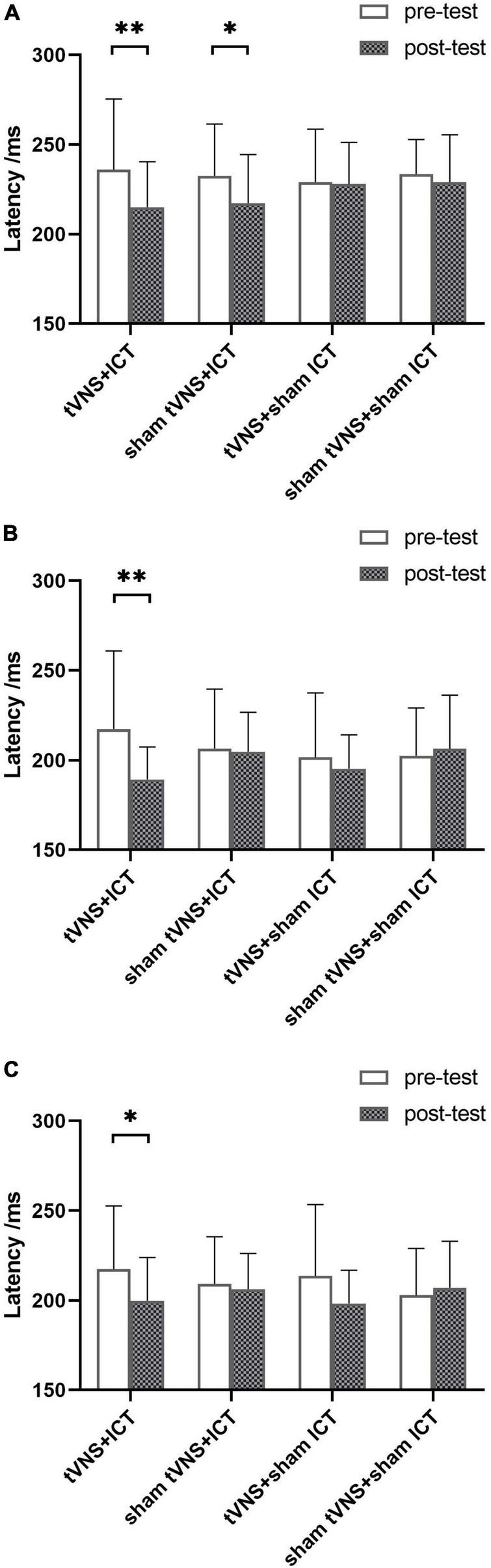
Summary of signal-N2 latency in SST. **(A)** Signal-N2 latency between pre- and post-test for each group in frontal region. **(B)** Signal-N2 latency between pre- and post-test for each group in fronto-central region. **(C)** Signal-N2 latency between pre- and post-test for each group in central region. Error bars represent the standard deviation of the mean. *p < 0.05, ^**^p < 0.01.

**FIGURE 3:**
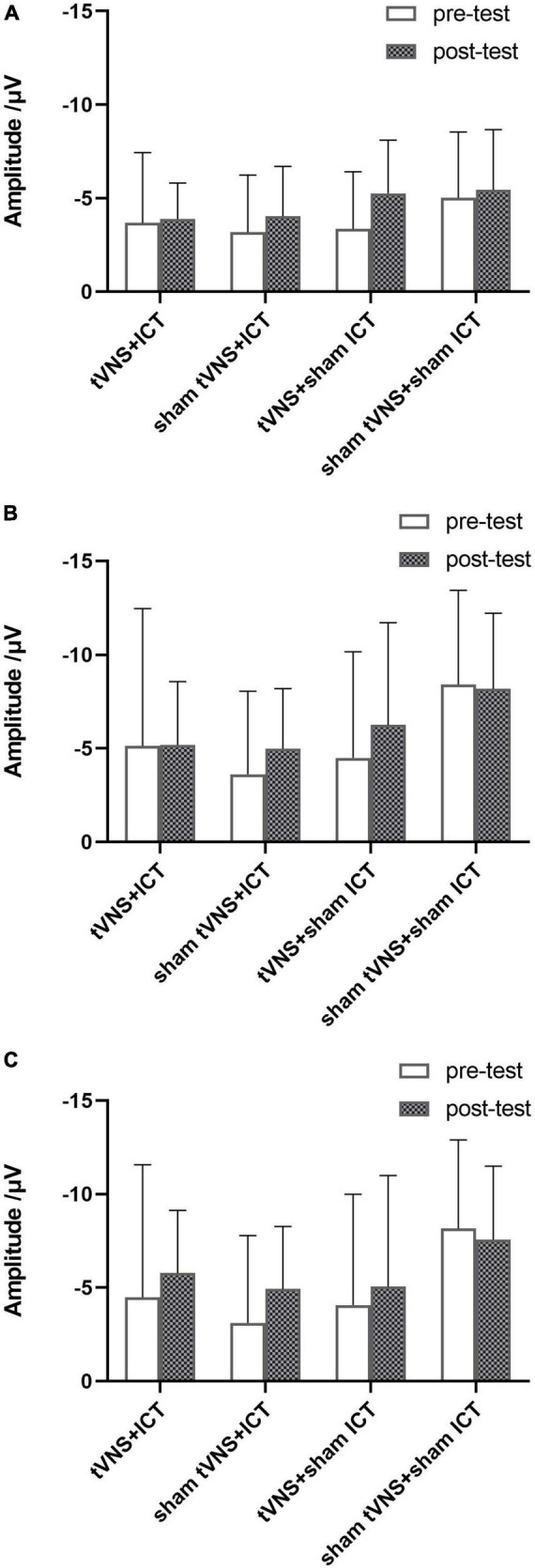
Summary of signal-N2 amplitude in SST. **(A)** Signal-N2 amplitude between pre- and post-test for each group in frontal region. **(B)** Signal-N2 amplitude between pre- and post-test for each group in fronto-central region. **(C)** Signal-N2 amplitude between pre- and post-test for each group in central region. Error bars represent the standard deviation of the mean.

ERP are depicted in Figures 2, 3. The results of simple effect using *post hoc* Bonferroni tests indicated that the tVNS + ICT group had significantly shorter signal-N2 latency at post-test than pre-test in frontal, fronto-central, and central regions (frontal: *p* = 0.007; fronto-central: *p* = 0.001; central: *p* = 0.044). Additionally, the sham tVNS + ICT group exhibited a significantly shorter signal-N2 latency at post-test than pre-test in the frontal region (*p* = 0.041). However, there was no significant difference between pre- and post-test in the tVNS + sham ICT or sham tVNS + sham ICT groups in frontal, fronto-central, and central regions. Additionally, the sham tVNS + ICT group exhibited a significantly shorter signal-N2 latency at post-test than at pre-test in the frontal region (*p* = 0.04). However, there was no significant difference between pre- and post-test in the tVNS + sham ICT or sham tVNS + sham ICT groups in frontal, fronto-central, and central regions.

### 3.3. Alpha power

As shown in Table 2, the rmANOVA of alpha power based on PSD revealed significant main effects of phase and group and a phase × group interaction effect in the ROIs.

**FIGURE 4:**
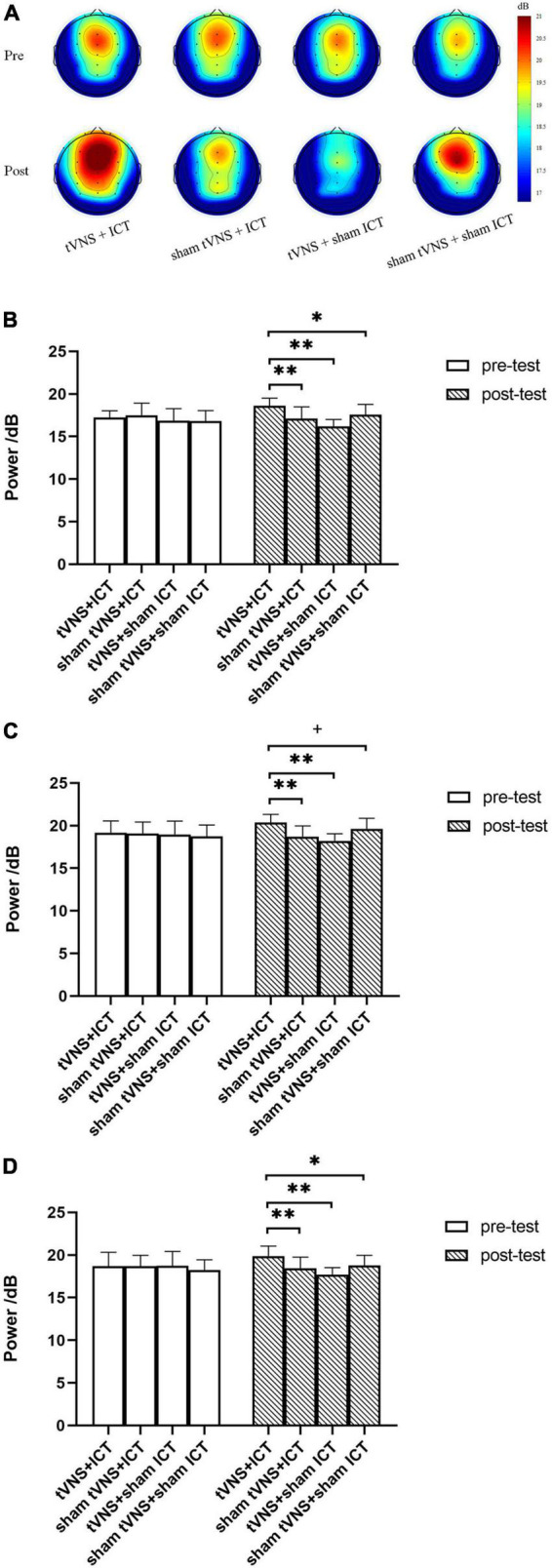
Summary of alpha power in SST. **(A)** Topography of alpha power spectrum density between the four groups during pre- and post-test phases. **(B)** Alpha power between groups, during pre- and post-test phases in frontal region. **(C)** Alpha power between groups, during pre- and post-test phases in fronto-central region. **(D)** Alpha power between groups, during pre- and post-test phases in central region. Error bars represent the standard deviation of the mean. ^**^p < 0.01, *p < 0.05, +*p* < 0.08.

To further explore the intervention effects between groups, we performed a simple-effect analysis using *post-hoc* analysis with Fisher’s LSD correction (Figure 4). We found no significant difference in alpha power between the four groups at baseline (in the pre-test phase). However, there was a significant difference in alpha power between the four groups in the post-test phase. The post-test results showed a significantly larger alpha power in the tVNS + ICT group than the sham tVNS + ICT group (frontal: *p* < 0.001; fronto-central: *p* < 0.01), the tVNS + sham ICT group (frontal: *p* < 0.001; fronto-central: *p* < 0.001), and the sham tVNS + sham ICT group (frontal: *p* = 0.01; fronto-central: *p* = 0.01) in frontal and central regions. Additionally, we found a significantly larger alpha power value in the tVNS + ICT group than in the sham tVNS + ICT (*p* < 0.001) and tVNS + sham ICT (*p* < 0.001) groups, and marginally significantly larger alpha power in the tVNS + ICT group than in the sham tVNS + sham ICT group (*p* = 0.077) in the fronto-central region.

## 4. Discussion

In the present study, we investigated whether tVNS and ICT exert a synergistic effect on IC enhancement and further investigated the enhancement effect in healthy adults according to cortical electrophysiological performance in selected ROIs. To our knowledge, this is the first study to demonstrate the synergistic effect of tVNS combined with ICT on electrophysiological brain activity in healthy adults. As hypothesized, we found that signal-N2 latency was significantly shorter and alpha power was significantly greater after the combined intervention with tVNS and ICT. Concerning the N2 component results, the tVNS + ICT and sham tVNS + ICT groups exhibited a shorter signal-N2 latency in the frontal region, which indicated that the timing of conflict monitoring in response inhibition was faster in healthy adults who had received ICT than in those who had not. This finding is consistent with those of previous studies on IC practice manifesting the frontal N2 latency modulation on the temporal dynamics (Millner et al., 2012), and subjects exhibited faster N2 latency after inhibition training (Schroder et al., 2020). In addition, our findings on the signal-N2 latency in the frontal region are in line with the effects of ICT on behavioral performance, whereby subjects who have undergone ICT obtained a faster stop-signal reaction time (Wang et al., 2022).

Furthermore, the signal-N2 latency was also shorter after the combined tVNS with ICT intervention in fronto-central and central regions, and this effect was not seen in the sham tVNS or sham ICT groups. This is consistent with the hypothesis that tVNS exerts a positive synergistic effect on IC enhancement, which, in turn, produces an accelerating effect of automatically generated response tendencies (Grützmann et al., 2022). This has been corroborated by findings from Pihlaja et al. (2020) who suggested that a change in the frontal N2 component is a neural marker of cognitive control.

By contrast, the signal-N2 latency did not differ significantly between the pre- and post-tests in the tVNS + sham ICT intervention group. tVNS has shown potential for benefiting cognition in healthy adults, such as modulating conflict monitoring in IC processes (Pihlaja et al., 2020). However, it is reportedly difficult to maintain the modulatory effect once tVNS has stopped (Van Leusden et al., 2015), which could explain why we found no difference in signal-N2 latency after the tVNS + sham ICT intervention.

Interestingly, we found no significant difference in signal-N2 amplitude between pre- and post-tests in the tVNS + ICT intervention group. In line with our previous results, where we found that *p*_(respond|_ _signal)_ in the SST did not differ significantly between the pre- and post-tests with tVNS + ICT intervention (Wang et al., 2022), the present finding on signal-N2 amplitude seems to demonstrate that N2 amplitude could be a marker with which to assess the ability of processing conflict signals monitored in IC. Additionally, the ceiling effect of response inhibition (Bürki et al., 2014) in the SST might be the main reason why there were no significant behavioral [*p*_(respond|_ _signal)_] or electrophysiological (signal-N2 amplitude) performance changes indicative of IC enhancement in the tVNS + ICT group.

For alpha power in the SST, the tVNS + ICT group obtained stronger alpha-band PSD compared with the sham tVNS + ICT, tVNS + sham ICT, and sham tVNS + sham ICT (placebo intervention) at the post-intervention phase in frontal, fronto-central, and central brain regions. Given that alpha power is thought to be associated with inhibition (Händel et al., 2011), the finding of larger alpha power after the tVNS + ICT intervention suggests that this combination can improve IC performance. Moreover, alpha oscillations have been linked to mechanisms underlying IC, such as the suppression of irrelevant or interfering information (Vaden et al., 2012). Our findings also support work showing a stronger power of frontal and central alpha oscillations when performance in inhibition tasks increases, via top-down inhibitory strategic processes (Hwang et al., 2016; Konjusha et al., 2022). Additionally, our findings on the change in alpha oscillations support reports that tVNS can activate endogenous neuromodulatory signaling, such as LC-NE activity, which is correlated with increased arousal (Sharon et al., 2021); this, in turn, improves the ability to overcome the interfering effects of irrelevant information in the prefrontal cortices.

One limitation of the present study was that we could not determine how the ability of tVNS + ICT to induce stronger alpha power in the selected ROIs influenced IC performance. We can speculate that the increased alpha power seen following the tVNS + ICT intervention may have a far transfer effect on cognitive functions (e.g., shift of attention task, multitasks in IC), including IC. Additionally, we did not investigate the optimal sessions of tVNS combined with ICT, which might produce greater IC enhancement. It is also unclear how long IC enhancement is maintained after the tVNS + ICT intervention. Accordingly, future research should focus on investigating the transfer effects, the optimal protocol, and the duration of the IC-enhancing effect of tVNS + ICT intervention. Although Results regarding alpha power in fronto-central region showed marginally significant difference between the tVNS + ICT group and the sham tVNS + sham ICT group, these results should be taken with caution. Thus, these findings with statistically marginal significance in present study also require further validation by expanding the sample size. Finally, due to the small sample size, the clinical implications of these findings in present study should explored by expanding the sample size in different participant populations in future research. To investigate the transfer effects and the optimal protocol of tVNS on brain activity, the evaluation of neural effects of tVNS by neuroimage techniques (e.g., TMS-EEG, fNIRs, fMRI) should be studied in future research.

## 5. Conclusion

In the present study, we demonstrated that tVNS combined with ICT shortens the signal-N2 latency and that increased alpha power in the SST is closely associated with IC enhancement. These findings provide neurophysiological evidence to suggest that tVNS combined with ICT may be a valuable method for enhancing IC, and may represent a novel and feasible approach for improving IC in adults with ADHD, those addicted to alcohol or drugs, people with obesity, and individuals who require a high inhibitory capacity, such as healthcare and military personnel, pilots, and astronauts.

## Data availability statement

The raw data supporting the conclusions of this article will be made available by the authors, without undue reservation.

## Ethics statement

The studies involving human participants were reviewed and approved by the Medical Ethics Committee of the Air Force Medical University. The patients/participants provided their written informed consent to participate in this study.

## Author contributions

CW, LZ, and ZW designed and conducted the study. XC, HW, LY, and ZW contributed to the study design. CW, YQ, ZG, YL, and JD contributed to recruitment of participants, data collection, and data analysis. CW and LZ were responsible for writing of the manuscript. HW, LY, and ZW contributed to review the literature and interpret the results. All authors contributed to the article and approved the submitted version.

## Supplementary material

Supplementary Table 1 The original data of Figure 3A.
